# Differential expression of genes during recovery of *Nicotiana tabacum* from tomato leaf curl Gujarat virus infection

**DOI:** 10.1007/s00425-023-04182-4

**Published:** 2023-07-05

**Authors:** T. Namgial, A. K. Singh, N. P. Singh, A. Francis, D. Chattopadhyay, A. Voloudakis, S. Chakraborty

**Affiliations:** 1 Laboratory of Plant Breeding and Biometry, Department of Crop Science, Agricultural University of Athens, Athens, 11855 Greece; 2 Molecular Virology Laboratory, School of Life Sciences, Jawaharlal Nehru University, New Delhi, 110067 India; 3 Laboratory of Plant Molecular Biology, National Institute of Plant Genome Research, New Delhi, 110067 India

**Keywords:** Geminivirus, Begomovirus, Symptom recovery, Tobacco, Next-generation sequencing (NGS), Differential gene expression, Pathogenesis

## Abstract

**Main conclusion:**

*Nicotiana tabacum* exhibits recovery response towards tomato leaf curl Gujarat virus. Transcriptome analysis revealed the differential expression of defense-related genes. Genes encoding for cysteine protease inhibitor, hormonal- and stress-related to DNA repair mechanism are found to be involved in the recovery process.

**Abstract:**

Elucidating the role of host factors in response to viral infection is crucial in understanding the plant host–virus interaction. *Begomovirus*, a genus in the family *Geminiviridae,* is reported throughout the globe and is known to cause serious crop diseases. Tomato leaf curl Gujarat virus (ToLCGV) infection in *Nicotiana tabacum* resulted in initial symptom expression followed by a quick recovery in the systemic leaves. Transcriptome analysis using next-generation sequencing (NGS) revealed a large number of differentially expressed genes both in symptomatic as well as recovered leaves when compared to mock-inoculated plants. The virus infected *N. tabacum* results in alteration of various metabolic pathways, phytohormone signaling pathway, defense related protein, protease inhibitor, and DNA repair pathway. RT-qPCR results indicated that *Germin-like protein subfamily T member 2* (*NtGLPST*)*, Cysteine protease inhibitor 1-like (NtCPI), Thaumatin-like protein* (*NtTLP*)*, Kirola-like* (*NtKL*), and *Ethylene-responsive transcription factor ERF109-like* (*NtERTFL*) were down-regulated in symptomatic leaves when compared to recovered leaves of ToLCGV-infected plants. In contrast, the *Auxin-responsive protein SAUR71-like* (*NtARPSL*) was found to be differentially down-regulated in recovered leaves when compared to symptomatic leaves and the mock-inoculated plants. Lastly, *Histone 2X protein like* (*NtHH2L*) gene was found to be down-regulated, whereas *Uncharacterized* (*NtUNCD*) was up-regulated in both symptomatic as well as recovered leaves compared to the mock-inoculated plants. Taken together, the present study suggests potential roles of the differentially expressed genes that might govern tobacco’s susceptibility and/or recovery response towards ToLCGV infection.

**Supplementary Information:**

The online version contains supplementary material available at 10.1007/s00425-023-04182-4.

## Introduction

Members of the largest plant virus family of *Geminiviridae* pose alarming threats to agriculture and food security worldwide and are characterized by small circular single-stranded DNA genomes. One of the well-studied genera of *Geminiviridae* is *Begomovirus* where monopartite (DNA-A, approximately 2.7 kb in genome size) or bipartite (DNA-A and DNA-B, each about 2.6 kb in genome size) members exist (Rojas et al. 2005). Tomato leaf curl Gujarat virus (ToLCGV) belongs to the genus *Begomovirus* and is a monopartite begomovirus, however, it was found to be in association with DNA-B component under natural conditions (Chakraborty et al. 2003a, b). In case of bipartite begomoviruses, most of the proteins encoded by the DNA-A component are associated with viral replication, whereas the DNA-B component regulates the movement of the virus. Monopartite begomoviruses, having only one DNA component, with their genome similar to the DNA-A component of the bipartite, encode proteins required for both replication and movement. In case of ToLCGV, association of DNA-B, isolated from the same infected tomato plant increases symptom severity (Chakraborty et al. 2003b, 2008). Begomoviruses have six to eight ORFs that are transcribed from both the complementary (C-strand) as well as the virion strand (V-strand) of the virus genome. The replication-associated protein (Rep/AC1/C1), the transcription activator protein (TrAP/AC2/C2), the replication enhancer protein (REn/AC3//C3), and AC4 are encoded in the C-stand, while the coat protein (CP/AV1/V1) and the pre-coat protein (AV2/V2) encoded by ORFs are present in the V-strand (Padidam et al. 1996). Additional ORF encode for C5 protein was reported recently from tomato yellow leaf curl virus (TYLCV) and was found to act as virulent factor and RNA silencing suppressor. The inhibition of viral replication and disease development in mutation of C5 TYLCV infected plant was also reported (Gong et al. 2021; Zhao et al. 2022). Experimental evidence about these proteins related to their structure and functions, and various host-associated partners have been described (Fondong 2013). Many of these proteins were found to be multifunctional, i.e., required for various events during the process of infection including RNA silencing (Devendran et al. 2022) and they co-evolved with host proteins, contributing to successful pathogenesis.

The evolutionary evidence suggests that there is a continuous arms race between the host and the invading virus. The virus could evolve efficiently using the host factors for its replication, overcoming plant defense, and causing symptoms in a permissive host. In contrast, plant defense against viral infection could lead to symptom recovery as means of resistance. CP interacts with host proteins such as importin α and karyopherin α1 to facilitate the nucleo-cytoplasmic shuttling of the viral genome (Kunik et al. 1999; Guerra-Peraza et al. 2005). CP also determines insect-vector specific transmission of the virus and specific sequence alteration result in change in insect transmission of the virus (Pan et al. 2020). Pre-coat protein acts as RNAi silencing suppressor (Zrachya et al. 2007) and pathogenicity determinant in many geminiviruses. The suppression of RNAi silencing by TYLCV-V2 required its interaction with SlSGS3 (suppressor of gene silencing 3) (Glick et al. 2008). Pre-coat protein conserved domain’s protein kinase C determines the pathogenicity of East African cassava mosaic Cameroon virus (EACMCV) (Chowda-Reddy et al. 2008). The TYLCV AV2 protein interacts with the host factor, CYP1, a papain-like cysteine and thus inhibit the HR response (Bar-Ziv et al. 2015). This protein blocks the host recovery process in tobacco plants infected with tomato leaf curl New Delhi virus (ToLCNDV) (Basu et al. 2018). AC1, encoding replication-associated protein (Rep), interacts with diverse host proteins such as the proliferating cell nuclear antigen (PCNA), the replication factor C (RFC), the replication protein A (RPA), the retinoblastoma related protein (RBR), and the SUMO-conjugating enzyme (SCE-1) (Rizvi et al. 2015; Ruhel and Chakraborty 2019). Rep protein from chilli leaf curl virus (ChiLCV) hijacks host ubiquitin machinery resulting in enhancement of the viral gene transcription (Kushwaha et al. 2017). Reduction of global cytosine methylation was reported from TrAP overexpressing transgenic plants (Buchmann et al. 2009) and it was the first protein identified from begomovirus to have silencing suppressor activity (Voinnet et al. 1999)**.** TrAP protein from TYLCV was reported to interact with a ubiquitin precursor protein, ribosomal protein S27A (RPS27a) and thus hindered the jasmonate ZIM-domain (JAZ) polyubiquitination in tobacco plants infected with TYLCV. The MYC2 transcription factor, which participates in transactivation of terpene-biosynthesis gene, inhibits the RPS27A-silenced plants or C2 transgenic lines, hence results in enhancement of the whitefly vector performance. This suggests the indirect mutual relationship between the virus and its vector (Li et al. 2019). MYC2 protein was also found to interact physically with nuclear shuttering protein (NSP) from cabbage leaf curl virus (CaLCuV), thus it has been implicated in the suppression of plant immune response by virus to favor the insect vector’s performance (Li et al. 2014).

REn protein interacts with geminivirus Rep thus enhance the viral DNA replication and symptom development (Sunter et al. 1990; Etessami et al. 1991). This observation is supported by recent discovery of REn protein recruiting DNA polymerase α and δ in TYLCV-infected *N. benthamiana* suggested its role in viral replication (Wu et al. 2021). In fact, REn enhances in vitro ATPase activity of Rep and also interacts with host factors including PCNA, RBR (Settlage et al. 1996, 2001, 2005) and with the transcription factor NAC1 result in increased the expression of NAC domain protein and thus enhances viral replication (Selth et al. 2005).

Different plant hosts infected by C4 mutant of tomato leaf curl virus (ToLCV) resulted in symptom reduction without affecting the viral accumulation (Rigden et al. 1994). The cell-cycle-associated proteins such as the cyclins cyc1, cyc2, and cyc2b, the cyclin-dependent kinases (CDK), and the cyclin-activated kinases (CAKs) levels are found to be elevated in *A. thaliana* expressing BSCTV C4 protein. The transgenic plants expressing C4 and BSCTV infection were found to suppress some cell cycle inhibitors (Park et al. 2010). Furthermore, recent reports suggest its ability to suppress SA-mediated defense (Medina-Puche et al. 2020) and HR (Mei et al. 2020) as well as confer drought stress tolerance in plants (Corrales‐Gutierrez et al. 2020). The C4 protein of TYLCV was shown to block systemic silencing by interacting with host *BARELY ANY MERISTEM 1*(BAM1) (Rosas-Diaz et al. 2018) and it also blocks the cell–cell movement of siRNAs, thus its role in silencing suppressor activity (Carluccio et al. 2018). In the absence of C4 protein, the other proteins such as TrAP, AV2, or Rep might take over its function, so it is dispensable and performs an obligatory function.

With the advancements in transcriptome analysis, using next-generation sequencing, the molecular complexity of host–virus interactions could be elucidated. Generally, RNA sequencing (RNAseq) is the most widely used approach for transcriptome analysis (Mosa et al. 2017). The previous study of recovery process in pepper plants infected with pepper golden mosaic virus (PepGMV) resulted in differential expression of several genes encoded for protein involved in plant defense response (pathogenesis-related proteins, hormone biosynthesis, and ROS production) (Góngora-Castillo et al. 2012). In addition, another study revealed differential expression of genes, when two varieties of tomato (resistant and susceptible) were infected with TYLCV (Chen et al. 2013). It is well established that viral infection of plants results in transcriptional reprogramming in the host plant (Hanley-Bowdoin et al. 2004; Louis and Rey 2015; Zanardo et al. 2019). It is relevant to mention here that tobacco plants inoculated with ToLCGV recovers from symptom at the later stage of infection (Basu et al. 2018), however, the molecular identification of host factors is not yet carried out. Therefore, RNAseq analysis was performed to identify differentially expressed host factors of tobacco infected with ToLCGV.

## Materials and methods

### Experimental plants and growth conditions

Tobacco (*N. tabacum*) cv. Xanthi plants were grown in soilrite (Prakruti Products Pvt. Ltd., Karnataka, India) and pots were maintained under controlled conditions with 25 ± 2 ℃ temperature, 60% relative humidity and 16/8 h light/dark photoperiod regime in an insect-proof greenhouse at School of Life Sciences, Jawaharlal Nehru University, New Delhi, India.

### Preparation of viral inoculum and infection

Three to four-week old *N. tabacum* plants (4-leaves stage) were infected with the partial tandem repeat constructs of ToLCGV cognate components [DNA-A (AY190290) and DNA-B (AY190291)] (Padidam et al. 1995; Ranjan et al. 2013). The inoculation is accomplished through infiltration of *Agrobacterium tumefaciens* strain EHA105 harboring viral constructs of ToLCGV (Chakraborty et al. 2008), where equimolar concentration of DNA-A and DNA-B was mixed and the mixture was used for agro-inoculation. The two uppermost leaves of each tobacco plant were inoculated with a 20 μl of *Agrobacterium* culture mixture followed by pricking with needle. A total of 12 plants were agro-inoculated for each ToLCGV infection as well as the mock plants. Plants inoculated with *A. tumefaciens* strain EHA105 carrying only pCAMBIA2300 were considered as mock plants.

### Total RNA isolation, RNAseq

Leaf samples (300–500 mg) were harvested from three biological replicates at 21 dpi and immediately snap-frozen into liquid nitrogen and stored in – 80 ℃ till further use. Total RNA was isolated from the plant material with TRIzol reagent (Sigma, USA) as per the manufacturer’s instructions. RNA quantity and quality were checked by OD measuring and agarose gel-electrophoresis (1.5%). RNA isolated was sent for RNA sequencing by Fasteris (Fasteris SA, Switzerland) on a HiSeq 4000 according to the in-house RNAseq protocol.

### Identification and analysis of DEGs by bioinformatics analysis of the RNAseq data

The obtained paired-end reads were filtered for adapter sequences as well as for low quality reads using NGSQC Toolkit (v2.3.3). The filtered reads were then mapped to the reference transcriptome of *N. tabacum* (GCF_000715135.1_Ntab-TN90) employing bwa (v0.7.17) (Li and Durbin 2009) using the default parameters. Subsequently, the mapped reads were counted for overlaps with transcripts using HTSeq-count (v0.11.1) (Anders et al. 2015) and the differential expression analysis was performed by the DESeq package (Anders and Huber 2010). This R package uses a count-based approach, and employs the negative binomial distribution to account for both biological and technical variability. Finally, differentially expressed genes (DEGs) were selected by applying a cut-off of *p* value ≤ 0.05, FDR ≤ 0.05 and log_2_ fold change ≥  + 1 and ≤  − 1. To identify viral transcripts within the transcriptome, we employed a two-step approach. Firstly, we performed BWA mapping using the reference sequences of ToLCGV genome (DNA-A, Gen Bank accession no AY190290 and DNA-B, GenBank accession no AY190291). This mapping allowed us to align the sequencing reads to the viral reference sequences. Subsequently, we utilized htseq-count to quantify the read count for each identified viral transcript. By employing this methodology, we were able to identify and quantify viral transcripts present in the transcriptome accurately.

### Functional classification of DEGs by gene ontology

The Kyoto Encyclopedia of Genes and Genomes (KEGG) and the Eukaryotic Orthologous Groups of proteins (KOG) annotation provide insight into the biological and functional aspect of the DEGs. The protein sequences were assigned to a KEGG pathway using the KEGG Annotation Server (KAAS, https://www.genome.jp/kegg/kaas/) by BLAST against the manually curated KEGG gene database. The proteins were clustered into 4825 KOGs that constitute 25 molecular protein families. The plot function in R is used to construct a scatter plot (volcano) representation of gene expression levels.

### Selection of differentially expressed genes for RT-qPCR validation

The top 50 up-regulated and 50 down-regulated genes in the tobacco-ToLCGV interaction were selected on the basis of their log_2_-fold change. Further selections of genes were narrowed down to eight in number based on available information about their biological role in plant response to biotic and abiotic stresses (Table 1).

**Table 1:** List of selected genes for RT-PCR and their functions

Accession numbers	Name of gene	Function	References
XM_016578910.1	*Germin-like protein subfamily T member 2*	*Germin-like protein* (GLP) gene called *CchGLP* and found to provide resistance against pepper golden mosaic virus (PepGMV) and pepper huasteco yellow vein virus (PHYVV)	(Mejía-Teniente et al. 2015)
XM_016603024.1	*Cysteine protease inhibitor 1-like*	Cystatins conferred resistance against insect predators and fungal infection	(Irie et al. 1996) (Ball et al. 1991)
XM_016594454.1	*Thaumatin-like protein*	These proteins are classified as pathogenesis related-5 (PR-5). PR-5 overexpressed in tomato *Sw-*7 resistance showed enhanced resistance to tomato spotted wilt tospovirus	(Singh et al. 1987; Ruiz-Medrano et al. 1992; Padmanabhan et al. 2019)
XM_016656857.1	*Kirola-like*	Kirola (a kiwifruit allergen) belongs to the major latex proteins (MLP) family based on sequence similarity	(Chen and Dai 2010; Malter and Wolf 2011)
XM_016636648.1	*Ethylene-responsive transcription factor ERF109-like*	Ethylene-responsive element-binding factors (ERF), are essential transcription factors involved in biotic and abiotic stress tolerance in plants	(Hao et al. 1998)
XM_016635370.1	*Auxin-responsive protein SAUR71-like*	SAUR71-like belongs to SAUR41 subfamily, and its expression was reported as responsive to the chloroplast’s function	(Bosco et al. 2004; Estavillo et al. 2011) and ABA signaling (Leonhardt et al. 2004; Zeng et al. 2012)
XM_016587673.1	*Histone H2AX-like*	Histone H2AX-like (H2AX) facilitates the orderly recruitment of other protein mediators of the DNA DSBs, inducing specific site repair	(Podhorecka et al. 2010)
XM_016606874.1	*Uncharacterized*	Function unknown	The present study

### RT-qPCR analysis

Each RNA sample was mixed with the denaturing solution composed of MOPS buffer, formaldehyde, formamide and then EtBr and loading dye. The RNA sample and denaturing mixture were mixed in the ratio of 1:3 and short-spined followed by heating at 72 °C (10 min), snap-chilled on ice and loaded on the agarose gel (1.5%) to check the quality of isolated RNA. The isolated RNA was used to examine the relative expression of various transcripts by RT-qPCR (Parkash et al 2020). *Actin* (EU938079.1) from *N. tabacum* was used as the internal control. The details of the primers used are provided in Table S1. Prism 9 software (GraphPad, San Diego, USA) was used to plot graphs.

### Statistical analysis

For each sample, three biological replicates were used and data were presented as mean values with standard error bars. Comparison between mean values was made using ANOVA *F* test as needed using Prism 9 software (GraphPad, San Diego, USA). Significance level used was *p* = 0.05.

## Results

### Disease development and transcriptome sequencing of tobacco (*N. tabacum*) inoculated with ToLCGV

**Fig. 1:**
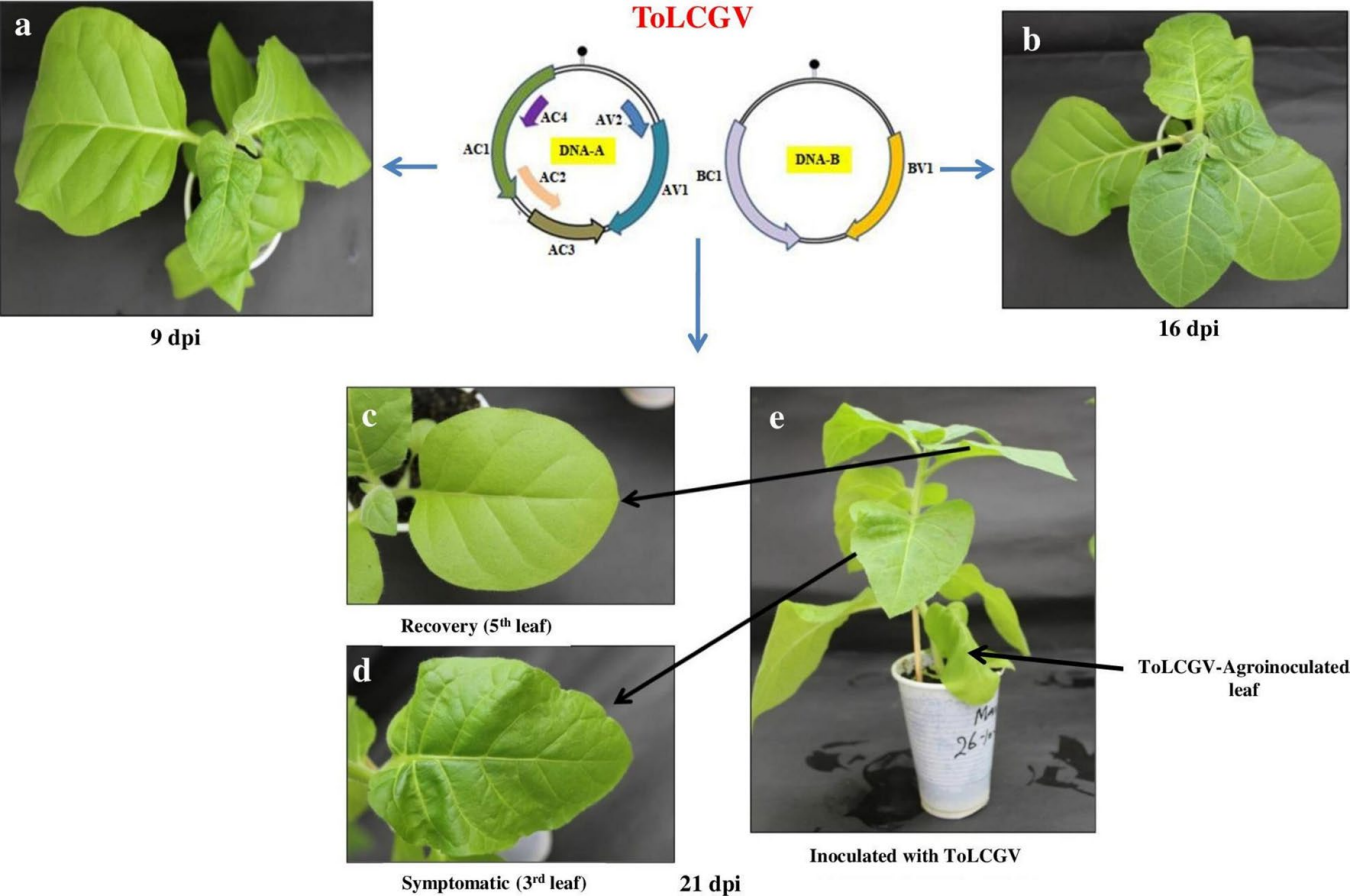
Tomato leaf curl Gujarat virus (ToLCGV) infection in tobacco plants: **a** At 9 dpi, plant exhibited severe symptoms followed by leaf curling and stunted growth; **b** At 16 dpi, plant started exhibiting recovery in symptoms; **c** and **d** showing recovery in leaf curling and leaf showing curling symptom at 21 dpi; **e** the plant having infected leaf as well as recovery in leaf symptom at 21 dpi. *dpi*: Days post inoculation

Mock plants, inoculated with the *Agrobacterium* harboring the empty vector, remained asymptomatic. Tobacco plants infected with ToLCGV (DNA-A + DNA-B) initially exhibited symptoms at 9 dpi followed by a quick recovery (5th leaf onwards) at 21 dpi; symptoms (mild curling, chlorotic spots on leaf lamina and altered leaf morphology, severe mosaic and vein banding) in the 3rd leaf persisted (Fig. 1). NGS was performed to identify the differential expression of genes in 3rd and 5th leaf of *N. tabacum.* Illumina paired-end sequencing resulted in a total of 412,783,560 reads from the three samples. After removing adapter sequences and discarding low quality reads, 404,147,432 (97.9%) reads were retained. These high-quality reads were further used to map on the reference *N. tabacum* (GCF_000715135.1_Ntab-TN90) using bwa. Approximately 87–94.9% of the reads were mapped to the *N. tabacum* genome. Detailed analyses of NGS including various percentage scores and GC content are shown in Table 2.

**Table 2:** Next generation sequencing data based on Illumina: data quality, filtration (clean reads), percentage, total mapped and percentage alignment summary for transcriptome analysis

Samples	Raw reads	Clean reads (filtered_reads)	GC (%)	Total mapped	% Mapped
Mock					
R1	69958320	68116636	44.26	129289502	94.90
R2	69958320	68116636	44.24		
VA + VB-S*					
R1	67526672	66284608	44.66	115397058	87.05
R2	67526672	66284608	44.7		
VA + VB-R**					
R1	68906788	67672472	43.76	127640472	94.31
R2	68906788	67672472	43.9		

*Symptomatic tobacco leaf tissues infected with ToLCGV

**Recovered tobacco leaf tissues infected with ToLCGV

The differential transcripts analysis was carried out to correlate viral gene expression with symptoms and recovery. It was observed that in symptomatic samples ToLCGV transcripts were clearly more abundant than the recovered tobacco leaves (Table 3).

**Table 3:** Comparative level of viral transcripts in symptomatic and recovered tobacco leaves infected with ToLCGV

Gene	CDS	Function	Symptomatic*	Recovered*
AC 1	AAO25671.1	Replication associated protein	17705	567
AC 3	AAO25669.1	Replication enhancer protein	1479	76
AV 1	AAO25668.1	Coat protein	549051	11022
AV 2	AAO25667.1	Pre-coat protein	26158	491
BC 1	AAO25674.1	Movement protein	13448	273
BV 1	AAO25673.1	Nuclear shuttle protein	71027	947

*Read count for each viral transcript in the symptomatic and recovered leaf samples

### ToLCGV-infected tobacco and differentially expressed genes

Interestingly, ToLCGV-infected tobacco plants initially exhibited symptoms (mild curling, chlorotic spots on leaf lamina and altered leaf morphology, severe mosaic and vein banding), starting from the third leaf, followed by a quick recovery (Fig. 1). A list of up- and down-regulated genes in symptomatic (3rd leaf) and recovered leaves (5th leaf) of ToLCGV-infected tobacco plants could be found in the Table 4.

**Table 4:** Differential expression of genes of tobacco plants infected with ToLCGV and their relative fold change

Gene ID	Gene name	Log_2_ fold change	
		Mock vs. VA/VB-S*	VA/VB-R** vs. VA/VB-S
XM_016578910.1	*NtGLPST*	− 7.1	− 6.7
XM_016603024.1	*NtCPI*	− 7.9	− 5.6
XM_016656857.1	*NtKL*	− 7.9	− 5.2
XM_016586295.1	*NtTKL*	− 5.2	− 3.6
XM_016635370.1	*NtARPSL*	− 5.8	4.5
		Mock vs. VA/VB-R	VA/VB-R vs. VA/VB-S
XM_016636648.1	*NtERTFL*	6.4	− 3.2
		Mock vs. VA/VB-R	Mock vs.VA/VB-S
XM_016587673.1	*NtHH2L*	− 6.0	− 10
XM_016606874.1	*NtUNCD*	8.1	9.6

Log_2_ fold in the number of genes when mock compared with VA/VB-R and VA/VB-S of ToLCGV infected plants

Negative value indicates down-regulation of respective genes. Virus combinations are indicated

*Symptomatic tobacco leaf tissues infected with ToLCGV

**Recovered tobacco leaf tissues infected with ToLCGV

**Fig. 2:**
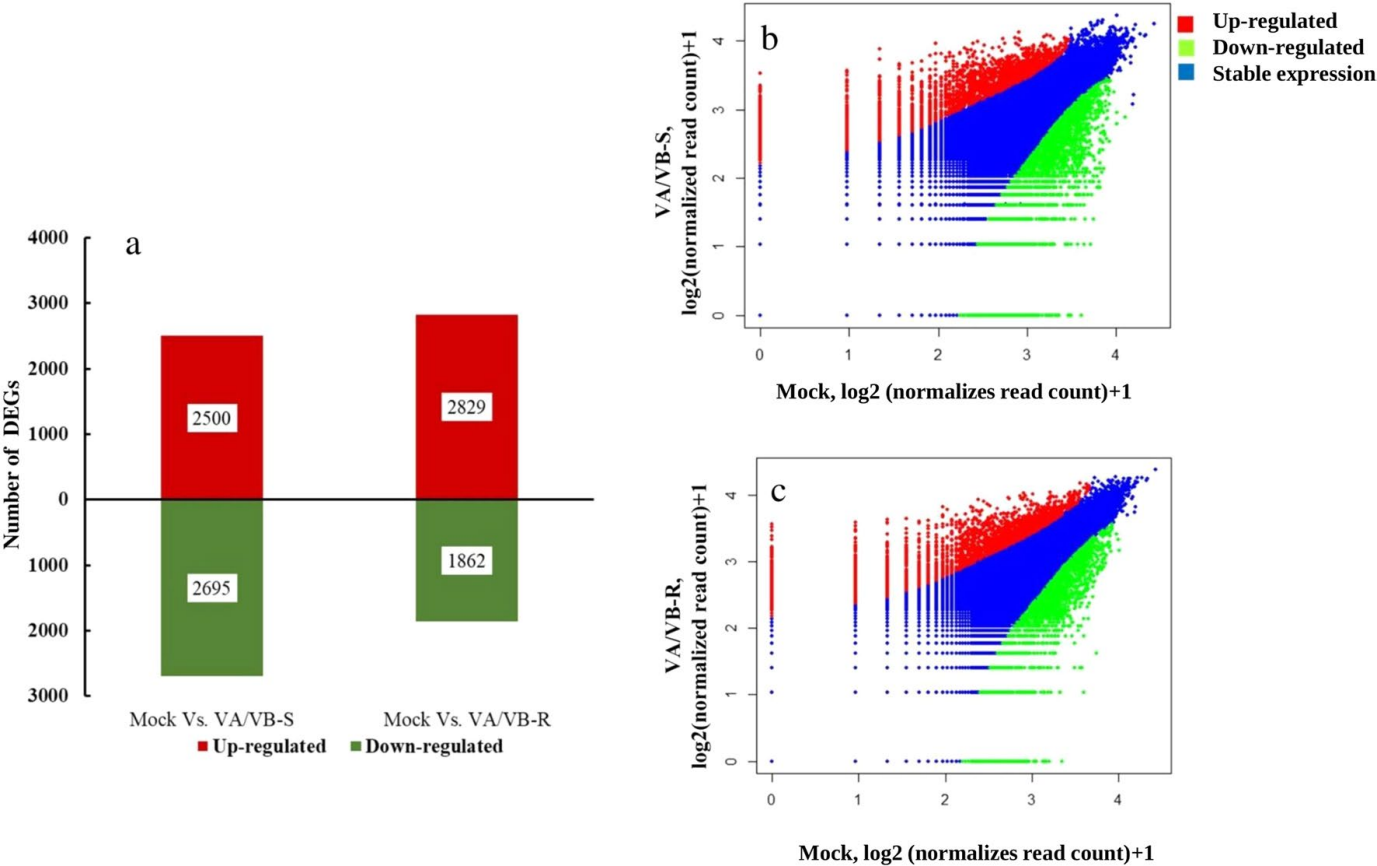
Differential expression of genes resulting from tomato leaf curl Gujarat virus infection (ToLCGV) in *N. tabacum*: **a** Bar graph showing the comparison of Mock vs. VA/VB-S (symptomatic leaf) revealed a total of 5195 differentially expressed genes, of which 2500 were up-regulated (Red color) and 2695 down-regulated (Green color). Comparison of Mock vs. VA/VB-R (recovered leaf) revealed 4691 differentially expressed genes, of which 2829 were up-regulated and 1862 were down-regulated. Volcano plot analysis of differentially expressed genes showed the up-regulated (Red color) and down-regulated (Green color) of genes in **b** VA/VB-S and **c** VA/VB-R when compared with Mock

**Fig. 3:**
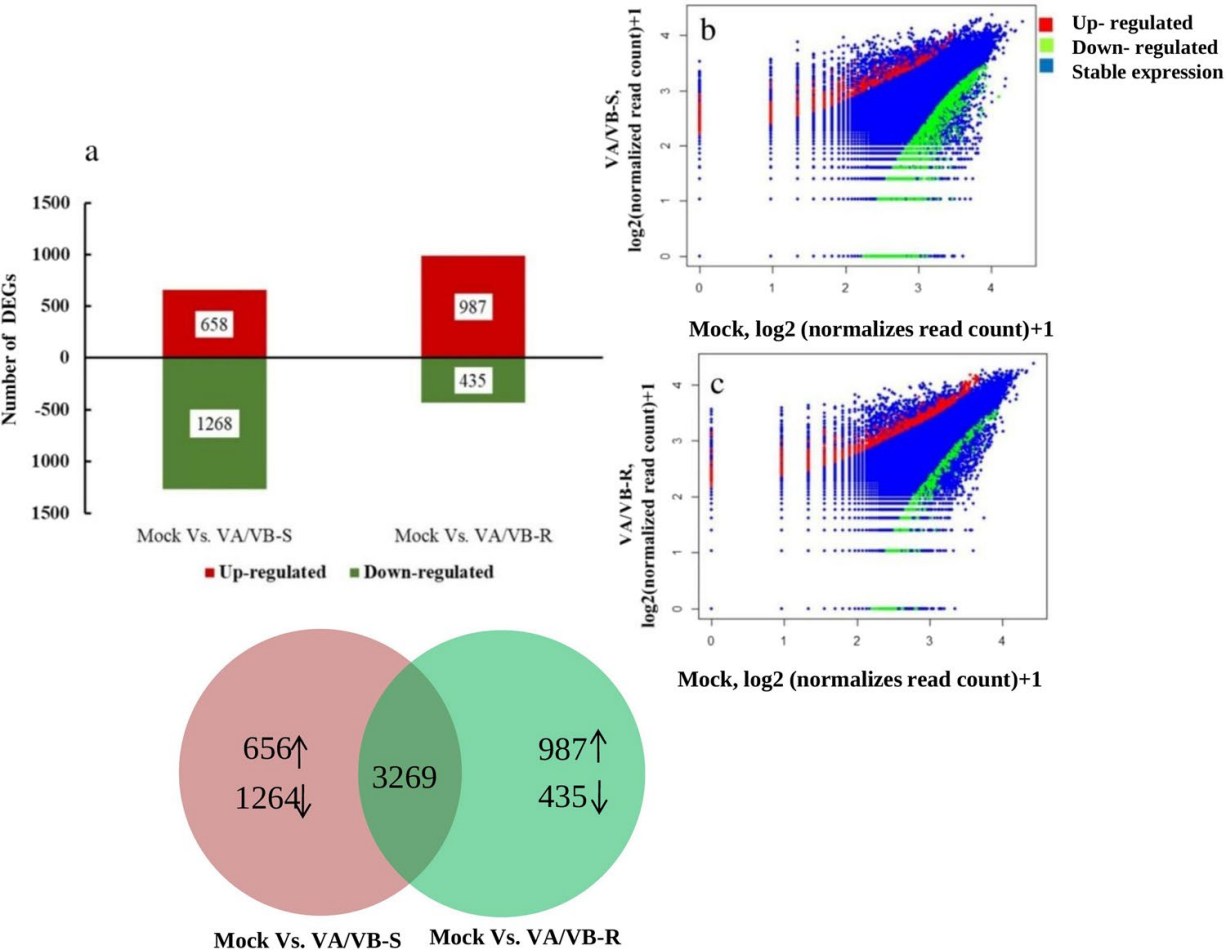
Transcriptome analysis of *N. tabacum* in response to tomato leaf curl Gujarat virus (ToLCGV) infection: **a** Comparison of Mock vs. VA/VB-S (symptomatic leaf) revealed a total of 1926 differentially expressed genes, of which 658 were up-regulated (Red color) and 1268 down-regulated (Green color). Comparison of Mock vs. recovered (VA/VB-R) (recovered leaf) revealed 1422 differentially expressed genes, of which 987 were up-regulated and 435 were down-regulated; Volcano plot showing the genes which were up-regulated (Red color) and were down-regulated (Green color) in **b** VA/VB-S and **c** VA/VB-R when compared with Mock **d** Venn diagram showing both the common and the uniquely DEGs in VA/VB-R and VA/VB-S when compared to mock

In total 5195 genes were differentially expressed in VA/VB-S tobacco plants as compared to mock plants (2500 up-regulated and 2695 down-regulated genes). Whereas, a total of 4691 genes were differentially expressed in VA/VB-R compared to the mock (2829 up-regulated and 1862 down-regulated genes). DEGs are shown in Fig. 2. We also analyzed the DEGs that were uniquely expressed in the VA/VB-S and VA/VB-R plants as compared to the mock-treated plants. Our analysis revealed that a total of 1926 genes were uniquely expressed in VA/VB-S plants as compared to the mock-inoculated plants, these DEGs include 658 up-regulated genes and 1268 down-regulated genes, where one third of the total genes were up-regulated and two-thirds of the genes were down-regulated (Fig. 3). However, VA/VB-R plants showed differential expression of 1422 unique genes, where most of the genes were up-regulated (987 up-regulated and 435 down-regulated genes). A total of 3269 genes were differentially expressed between VA/VB-S and VA/VB-R (Fig. 3).

### GO enrichment analysis

Gene ontology (GO) assignment was performed to classify the gene function of DEGs. DEGs can be categorized into three main categories: biological process, cellular component, and molecular function. The results were enriched in symptomatic and recovered leaves of VA/VB-inoculated plants; it was found in the biological process category: a large number of DEGs were related to response to stimulus, metabolic process, and primary metabolic process. In the cellular component category, the majority of DEGs were responsible for cellular anatomical entity and membrane. In the molecular function category, a high proportion of DEGs were related to catalytic activity and transferase activity. The cellular process and organic substance metabolic process belong to the biological process category and lipid binding from the molecular function category were enriched more in recovered leaves (Fig. S1).

**Fig. 4:**
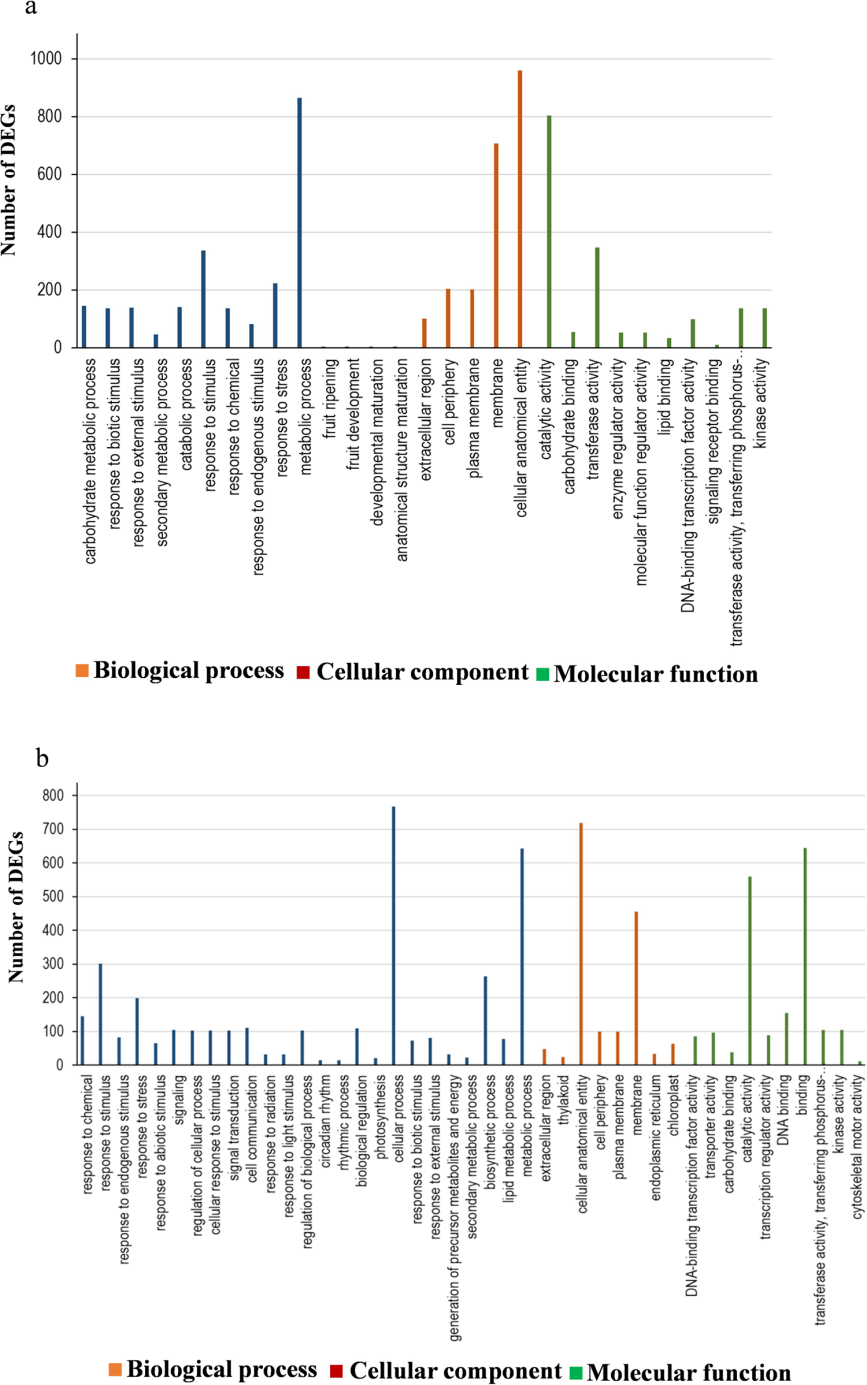
Functional classification of differentially expressed genes (DEGs) using Gene Ontology (GO). **a** function of uniquely expressed DEGs in VA/VB-S (symptomatic leaf) vs. mock-treated plants, **b** VA/VB-R (recovered leaf) plants vs. mock-treated plants

To further understand the DEGs in VA/VB-S and VA/VB-R plants, we also analyzed the gene ontology (GO) for the DEGs that were uniquely expressed in the VA/VB-S and VA/VB-R plants as compared to the mock-treated plants. Our analysis revealed that under the category of biological process a large portion of the DEGs belongs to the response to stimulus and metabolic process. In the cellular component category, the majority of the DEGs belong to the cellular anatomical entity and membrane group. In the molecular function category, a high portion of DEGs were related to catalytic activity process (Fig. 4a, b).

### Annotation of differentially expressed genes of *N*. *tabacum* in response to ToLCGV infection

**Fig. 5:**
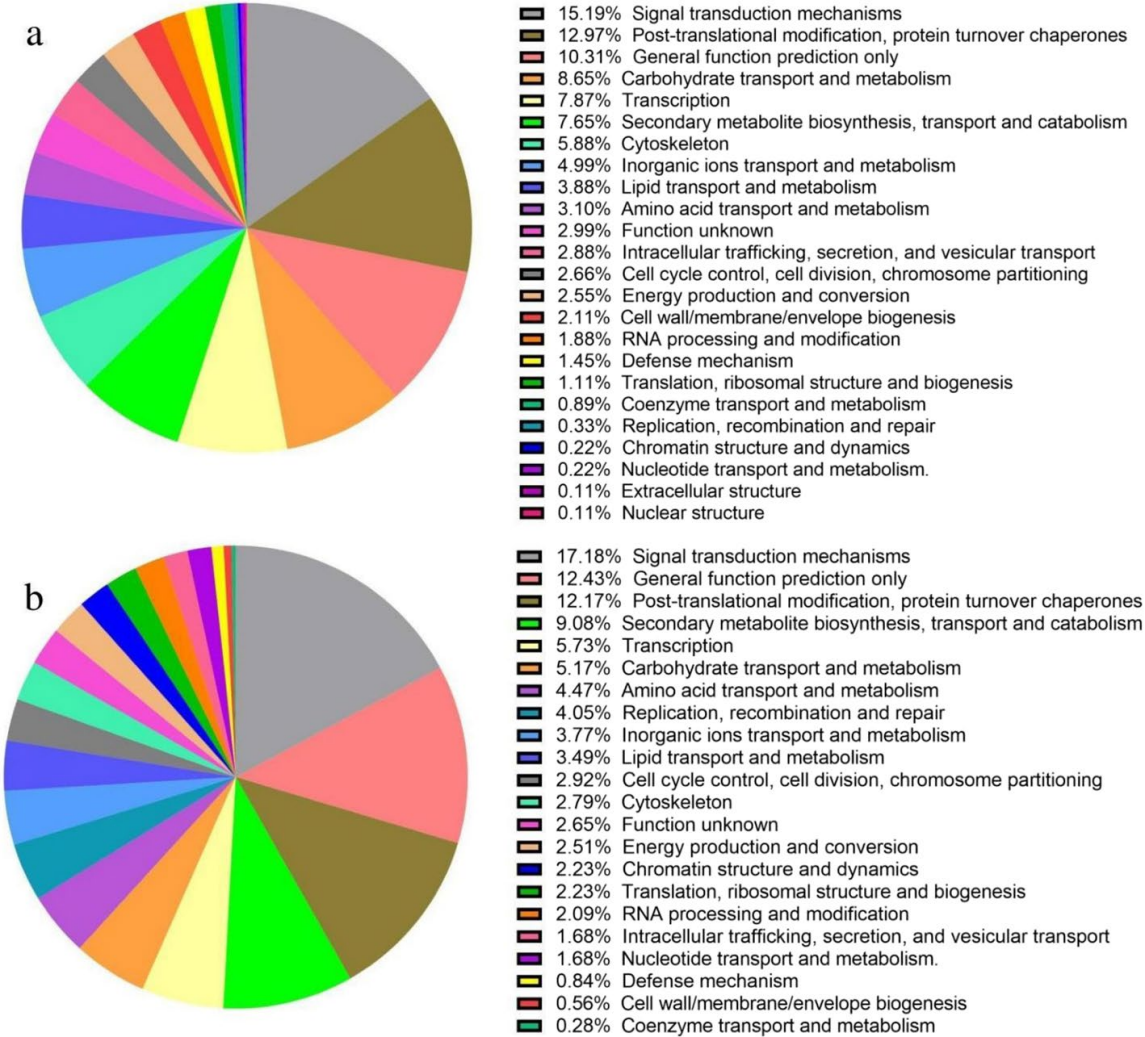
Annotation of differentially expressed genes of *N. tabacum* in response to tomato leaf curl Gujarat virus (ToLCGV): The KEGG and KOG annotation provides insight into the biological and functional aspect of the differentially expressed genes (DEGs). The pie charts represent the categorization and relative percentage of differentially expressed genes showing homology with known genes; **a** uniquely (DEGs) in VA/VB-R as compared to mock; **b** uniquely (DEGs) in VA/VB-S as compared to mock. *VA/VB-R*: ToLCGV-infected recovered leaves, *VA/VB-S*: ToLCGV*-*infected symptomatic leaves

The protein sequences were assigned to the KEGG pathway. The result from VA/VB-R when compared to mock plants, revealed that 15.19% of DEGs found to be involved in signal transduction mechanisms, whereas post-translational modification, protein turnover chaperones constituted 12.97%, followed by general function prediction (10.31%), transport and metabolism of carbohydrate (8.65%), inorganic ions (4.99%), lipids (3.88%), amino acid (3.10%), coenzyme (0.89%) and nucleotide (0.22%), transcription process (7.87%), secondary metabolite biosynthesis, transport and catabolism (7.65%), cytoskeleton (5.88%), function unknown (2.99%), intracellular trafficking, secretion, and vesicular transport (2.88%), cell cycle control, cell division, chromosome partitioning (2.66%), energy production and conversion (2.55%), cell wall/membrane/envelope biogenesis (2.11%), RNA processing and modification (1.88%), defense mechanism (1.45%), translation, ribosomal structure and biogenesis (1.11%). Similarly, other transcripts showed some degree of homology with genes involved in replication, recombination and repair (0.33%), chromatin structure and dynamics (0.22%), and nuclear and extracellular structure (0.11%) (Fig. 5a).

In contrast, when VA/VB-S was compared to mock inoculated plants, it was found that proteins encoded by DEGs exhibited considerable degree of homology with genes involved in different molecular processes such as signal transduction mechanisms (17.18%), post-transcriptional modification protein turnover, chaperones (12.71%), other general function (12.43%), secondary metabolites biosynthesis transport and catabolism (9.08%). transcription (5.73%), transport and metabolism of carbohydrate (5.17%), lipid (3.49%), nucleotides (1.68%), inorganic ion (3.77%), amino acids (4.47%), coenzyme (0.28%), replication, recombination and repair (4.05%), cytoskeleton (2.79%), with genes whose function is not known accounting to 2.65%, energy production and conversion (2.51%), cell division and chromosome partitioning (2.92%), chromosome structure and dynamics (2.23%), translation, ribosomal structure and biogenesis (2.23%), RNA processing and modification (2.09%), secretion and vesicular transport (1.68%), defense mechanism (0.84%), and cell wall/membrane/envelope biogenesis (0.56%) (Fig. 5b).

### Validation of selected differentially expressed genes via RT-qPCR

Following the DEG analysis, we selected a total of eight host genes based on their high fold change and available function information from symptomatic and recovered leaves from ToLCGV-infected plants to confirm their transcript expression level through RT-qPCR. These selected DEGs were: *Germin-like protein subfamily T member 2* (*NtGLPST*)*, Cysteine protease inhibitor 1-like (NtCPI), Thaumatin-like protein* (*NtTLP*)*, Kirola-like* (*NtKL*), *Ethylene-responsive transcription factor ERF109-like (NtERTFL)*, *Uncharacterized (NtUNCD), Auxin-responsive protein SAUR71-like* (*NtARPSL*), and *Histone 2X protein like* (*NtHH2L*).

**Fig. 6:**
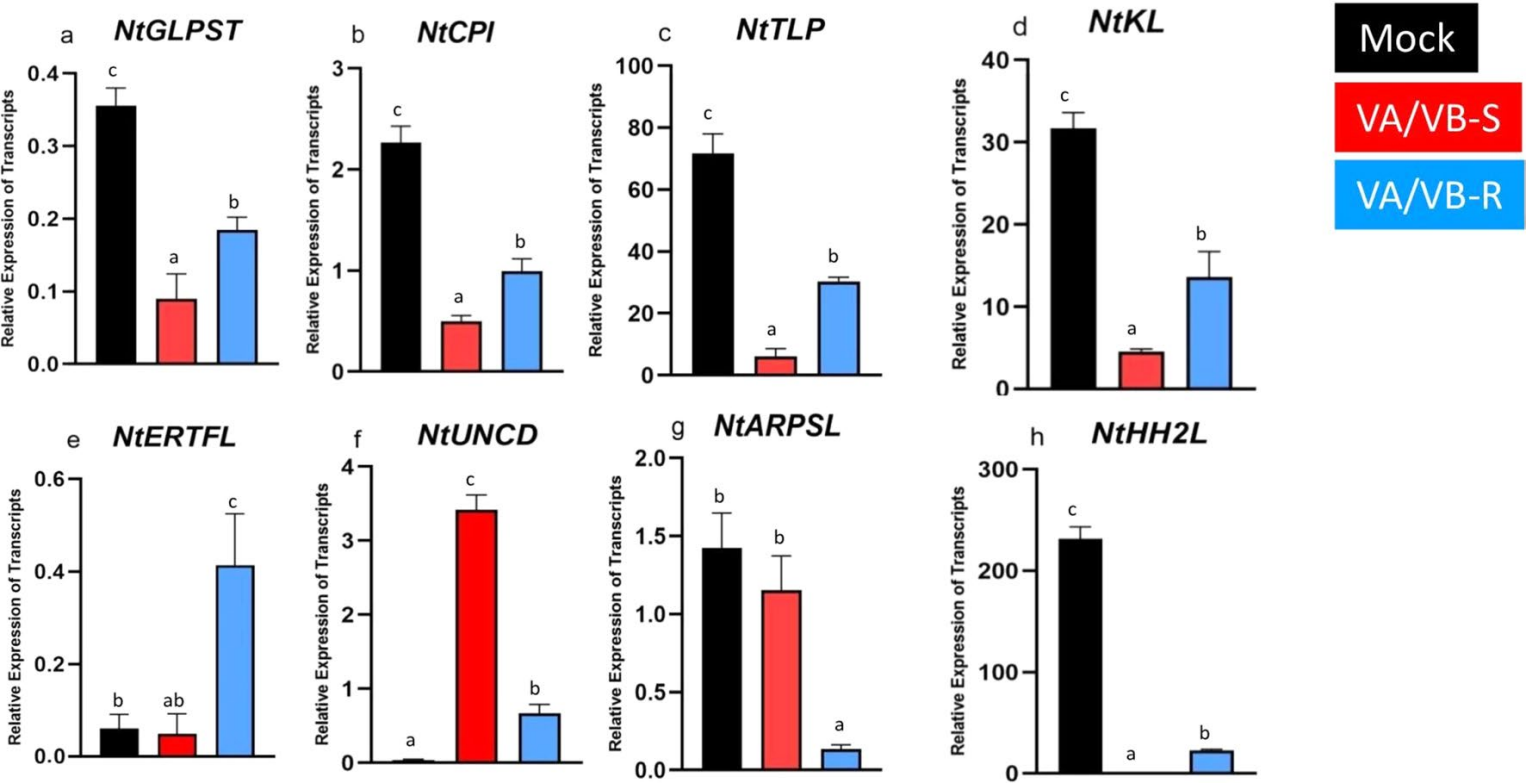
Begomovirus infection in *N. tabacum* and recovery in case tomato leaf curl Gujarat virus (ToLCGV) resulted in change in the transcripts level of the following genes estimated by RT-qPCR: **a** *Germin-like protein subfamily T member 2 (NtGLPST)*;** b** *Cysteine protease inhibitor 1-like(NtCPI)*; **c** *Thaumatin-like protein (NtTLP)*; **d** *Kirola-like (NtKL)*; **e** *Ethylene-responsive transcription factor ERF109-like (NtERTFL)*; **f** *Uncharacterized gene(NtUNCD)*; **g** *Auxin-responsive protein SAUR71-like (NtARPSL)*; **h** *Histone H2AX-like (NtHH2L).* The significant *P* value is denoted by letters. Three biological replicates were taken for each sample and *Actin* gene (EU938079.1) was used as the internal control

A large number of genes were differentially expressed in the ToLCGV-infected tobacco plants. NGS analysis revealed that the *Germin-like protein subfamily T member 2* (*NtGLPST*) was 6.7- and 7.1-fold down-regulated in VA/VB-S-infected plants as compared to VA/VB-R and mock plants (Table 3). RT-qPCR analysis also confirmed the down-regulation of *NtGLPST* in VA/VB-S plants (Fig. 6a). Similarly, expression of *Cysteine protease inhibitor 1-like* (*NtCPI*) was 5.6- and 7.9-fold down-regulated in VA/VB-S-infected tobacco plants as compared to VA/VB-R (Table 4) and the mock plants exhibited a decrease in gene expression levels in RT-qPCR analysis (Fig. 6b). NGS results showed that the *Thaumatin-like protein (NtTLP)* expression was 3.6- and 5.2-fold down-regulated in VA/VB-S plants (Table 4) and it was also verified through RT-qPCR (Fig. 6c). Expression of *Kirola-like* (*NtKL*) was also down-regulated in the VA/VB-S plants as compared to the VA/VB-R and mock plants and confirmed by RT-qPCR (Fig. 6d). NGS results showed 5.2- and 7.9-fold reductions of *NtKL* level in VA/VB-S when compared to VA/VB-R and mock inoculated plants (Table 4). The relative expression levels of *Ethylene-responsive transcription factor ERF109-like (NtERTFL)* result from RT-qPCR was consistent with the NGS data. NGS suggested 6.4 folds up-regulation in VA/VB-R when compared to mock plants, whereas 3.2 folds down-regulation in VA/VB-S compared to VA/VB-R (Fig. 6e). The RT-qPCR analysis suggested an up-regulation of *NtERTFL* in VA/VB-S when compared to VA/VB-R and no significant change of *NtERTFL* in VA/VB-S as compared to mock-inoculated plants.

Similarly, relative expression of the uncharacterized gene was highly expressed in VA/VB-S (ninefold) and VA/VB-R (sevenfold) as compared to the mock plants as shown by NGS, which is further confirmed by an increase in level of this gene in VA/VB-S and VA/VB-R (Fig. 6f).

The *Auxin-responsive protein SAUR71-like* (*NtARPSL*) was also differentially expressed in the ToLCGV-infected plants with NGS results showing a 5.8- and 4.5-fold down-regulation in the VA/VB-R plants as compared to the mock-treated and VA/VB-S plants, respectively (Table 4). RT-qPCR results also indicated low levels of *NtARPSL* in VA/VB-R as compared to both mock and VA/VB-S plants (Fig. 6g).

RT-qPCR of relative expression levels of *Histone 2X protein like (NtHH2L)* in VA/VB-S and VA/VB-R plants compared with mock-inoculated plant suggested that, *Histone 2X protein like (NtHH2L)* gene was found to be differentially expressed (Fig. 6h). The *NtHH2L* was 6- and tenfold down-regulated in both recovered and symptomatic leaves respectively when compared to mock (Table 4).

## Discussion

To unveil the host factors associated with plant antiviral defense and/or the plant proteins manipulated by viruses, it is crucial to study the plant host–virus interaction at the molecular level. The present study will provide insights on how viruses manipulate the host factors for their benefit and counteract plant virus by exhibiting disease recovery. The viral ORFs, low in number, encode for proteins that are essential for replication, movement, and viral transmission. To execute these functions, viruses interact with and recruit a number of host proteins and exploit many cellular pathways (Hanley-Bowdoin et al. 2013). Plants, in return, exhibit defensive antiviral strategies by recruiting host factors at various stages of infection to protect from viral invasion (Kumar 2019). In the present study, we have identified potential host factors which might either be involved in resistance/susceptibility to ToLCGV infection in tobacco. Further analysis of individual DEGs’ function is underway.

The result of functional enrichment analysis from VA/VB-S vs. mock plants and VA/VB-R vs. mock plants suggested that the DEGs belong to biological processes, cellular components, and molecular functions. The majority of DEGs belong to biological process followed by molecular functions group, and lastly by cellular components group (Fig. 4). DEGs involved in plant–virus interactions, which included: response to stimulus, metabolic process, primary metabolic process, cellular anatomical entity, membrane, catalytic activity, transferase activity, cellular process, biosynthetic process, organic substance metabolic process, and lipid binding, in agreement to an earlier study of Padmanabhan et al. 2019.

To gain a better understanding of the function of selected genes, we used the KEGG pathway analysis which revealed that, in both cases (symptomatic and recovery leaves), most DEGs belong to the signal transduction mechanisms, post-translational modification, protein turnover chaperones, and secondary metabolites biosynthesis transport and catabolism. Interestingly, we noticed that proteins in the categories of replication, recombination, repair, and structural dynamics of the chromosome were present in a higher percentage in the VA/VB-S (symptomatic leaves), implying that these structural and functional changes associated with DEGs in VA/VB-S resulted in symptom development. In contrast, the categories of transcription process, secondary metabolite biosynthesis, transport and catabolism, cytoskeleton, cell wall/membrane/envelope biogenesis, and defense mechanism-related DEGs were present in higher percentage in VA/VB-R, indicating their possible association with the recovery process. Importantly, changes in the structural dynamics of chromatin, nuclear and extracellular structure were observed only in the VA/VB-R.

The RT-qPCR results obtained correlated nicely with the NGS DEG data performed providing a validation of the NGS analysis. *Capsicum chinense* (BG-3821) transcriptional studies resulted in the identification of a *Germin-like protein* (GLP) gene called *CchGLP* and found to provide resistance against PepGMV and pepper huasteco yellow vein virus (PHYVV), both belonging to the family *Geminiviridae*. Transgenic *N*. *tabacum* (cv. Xanthi) expressing *CchGLP* exhibited resistance to geminivirus infections, making this gene an important candidate for geminivirus resistance (Mejía-Teniente et al. 2015). Our NGS analysis and subsequent RT-qPCR results revealed that *NtGLPST* is down-regulated in VA/VB-S (symptomatic leaf) when compared to VA/VB-R (recovered leaf) and the mock plants (Fig. 6a). This suggests that *NtGLPST* may act as a positive regulator of plant defense to begomovirus infection in tobacco.

Cysteine proteinase inhibitors (CPIs) (or cystatins), as antiviral agents, have been reported in many viruses for which cysteine proteinase is involved in their replication. Furthermore, cystatins conferred resistance against insect predators (Irie et al. 1996) and fungal infection (Ball et al. 1991). Additionally, cystatins were reported as hypersensitivity response (HR) cell death modulators in plant cells undergoing both abiotic and biotic stresses (Ye and Varner 1996; Ebel and Mithöfer 1998; Solomon et al. 1999). Expression of *Arabidopsis thaliana* cystatin (AtCYS1) in transgenic tobacco plant was reported to inhibit HR cell death induced by nitrous oxide and bacterial infection (Belenghi et al. 2003). Thus, the cysteine proteinase inhibitors are essential modulators of oxidative stress that cause PCD (programmed cell death) providing an antiviral role. In the case of the dianthovirus red clover necrotic mosaic virus (RCNMV), the RNA virus exploits the ROS generation during PCD for its replication (Hyodo et al. 2017). Hence, in the present study the observed down-regulation of *NtCPI* in VA/VB-S, when compared to VA/VB-R and the mock (Fig. 6b), suggesting that the begomovirus might be using PCD as the source of ROS for its own replication. Thus, the PCD regulation, by the expression of host *NtCPI*, could support viral replication. Since this kind of ROS-dependent viral replication has not been characterized in plants infected with DNA viruses, it is imperative to be the focus of future studies.

The comparison of the VA/VB-S with VA/VB-R and the mock plant indicated differential gene expression in the interaction of tobacco with ToLCGV. For example, *NtTLP* is down-regulated in symptomatic leaf (Fig. 6c), suggesting that *NtTLP* might have an important antiviral function. Thaumatins are structurally complex and correlated with plant cell’s osmotic adaptation. These proteins are classified as pathogenesis related-5 (PR-5) and are homologous to the osmotin like protein (OLP) and osmotin (Singh et al. 1987; Ruiz-Medrano et al. 1992). PR-5 overexpressed in tomato *Sw-*7 resistant line showed enhanced resistance to tomato spotted wilt tospovirus (Padmanabhan et al. 2019). Antifungal activity of OLP against *Phytophthora infestans* was reported (Zhu et al. 1996). It should be noted that tobacco mosaic virus (TMV) infection caused the overexpression of OLP and thaumatin in tobacco (Cornelissen et al. 1986). The study of recovery mechanism in pepper plants infected with PepGMV also suggested involvement of thaumatins in recovered pepper plants (Góngora-Castillo et al. 2012).

*NtKL* was found to be down-regulated in VA/VB-S when compared to VA/VB-R and mock plants and this could suggest that ToLCGV may down-regulate the expression of *NtKL* in order to cause infection (Fig. 6d). However, the function of NtKL has not yet been elucidated. Kirola (a kiwifruit allergen) belongs to the major latex proteins (MLP) family based on sequence similarity. The kiwelling ripening-related protein (RRP1) induction was observed in pepper plants infected with different viruses that suggested generalized role of RRP1 in a broad range of biotic stress defense mechanisms (Góngora-Castillo et al. 2012). There is sparse knowledge on e.g. kirola and kiwellin, although MLPs were reported to be overexpressed during viral and fungal infection, drought, and salt stress (Chen and Dai 2010; Malter and Wolf 2011). Thus, further study is needed to clarify the importance of *NtKL* in begomovirus infection in tobacco.

Ethylene-responsive element-binding factors (ERF), are essential transcription factors involved in biotic and abiotic stress tolerance in plants (Hao et al. 1998). The involvement of ERF in PCD was also reported (Bahieldin et al. 2016). It is found that the ERF109 gene responds to JA as well as to ethylene in Arabidopsis during biotic and abiotic stresses to maintain the redox homeostasis (Khandelwal et al. 2008; Kerchev et al. 2013). The anti-microbial chemical, named ningnanmycin (NNM) showed antiviral activity against TMV infection. The significant up-regulation of ERF109 in NNM-treated *N. tabacum* suggests its role in anti-viral defense (An et al. 2019). Transcriptome results suggested that ERF109 was up-regulated in recovered leaves when compared to mock, whereas it was down-regulated in symptomatic leaves when compared to recovered leaves (Fig. 6e). Therefore, this gene might act as a resistance factor in geminivirus pathogenesis that needs to be functionally validated.

*NtUNCD*, encoding an uncharacterized protein, is induced in both symptomatic as well as the recovery tissue, but it is induced more in the symptomatic leaves (Fig. 6f). Therefore, it is essential to characterize the protein encoded by this gene and determine its role and function related to the host-begomovirus interaction.

Auxin is a plant hormone that plays a significant physiological role in plant growth by sustaining apical dominance. Viral infection results in the loss of apical dominance (Kazan and Manners 2009). Primary auxin response genes consist of members of three gene families: Indole-3-Acetic Acid (I Aux/IAAs), SMALL AUXIN UP RNA (SAURs), and GRETCHEN HAGEN 3 (GH3s) (Hagen and Guilfoyle 2002). Although the function of the SAUR family is not well known, recent experiments in rice and *Arabidopsis* started shedding light on the function of the SAUR family proteins. SAUR71-like belongs to SAUR41 subfamily, and its expression was reported as responsive to the chloroplast’s function by inhibiting the non-photochemical quenching (Bosco et al. 2004; Estavillo et al. 2011) and ABA signaling (Leonhardt et al. 2004; Zeng et al. 2012). Due to the lack of knowledge about *SAUR71-like* involvement in plant-virus interaction, it is difficult to explain the observed repression of *NtARPSL* in VA/VB-R when compared to VA/VB-S and the mock plants (Fig. 6g), and thus further functional investigation is needed.

The DNA double-strand breaks (DSBs), which are fatal to the cell, need to be repaired for the cell to remain viable. It is reported that phosphorylation of Histone H2AX-like (H2AX) facilitates the orderly recruitment of other protein mediators of the DNA DSBs, inducing specific site repair (Podhorecka et al. 2010). Histone variant H2AX (γ-H2AX) functions in detection of host plant’s DSBs and DNA damage was reported in plant infecting microbes including fungi, bacteria, and oomycetes. The rapid phosphorylation of γ-H2AX in response to DNA damage is conserved across multicellular organisms (Rogakou et al. 1998; Iacovoni et al. 2010). DNA DSB damage remains a general feature of pathogenesis by pathogenic plant microbes. The γ-H2AX-associated repair of DNA DSBs maintains the genome integrity of the plant (Song and Bent 2014). It was earlier reported that some factors involved in plant DNA DSBs repair are regulated by geminivirus (i.e., cabbage leaf curl virus) infection (Ascencio-Ibánez et al. 2008). The up-regulation of such factor retards the geminivirus infection (Richter and Jeske 2015). In the present study, the down regulation of *NtHH2L* in symptomatic leaves suggested that geminivirus infection overcomes the plant defense through DNA DSBs repair mechanism for successful pathogenesis, requiring further investigation.

## Conclusion

The present study reveals the transcription activation and repression of a number of genes that could participate in the plant-begomovirus infection. The viral transcript analysis also suggested downregulation of ToLCGV transcription in recovered leaves. Hence, the tobacco genes differentially regulated following ToLCGV infection might play important roles in improving the protection against the viral infection, which needs to be further experimentally validated. The recovery phenotype in the ToLCGV–tobacco interaction, depicting plant’s ability to overcome the viral infection, might be influenced at the transcriptional level of a number of genes and the thorough understanding of the plant’s response could allow scientists to shift the host–virus interaction towards the exhibition of resistance in crops for the benefit of the farmer leading to a sustainable agriculture.

### *Author contribution statement*

TN, AV, and SC conceived and designed the research. NPS, AF, and DC performed the bioinformatic analysis. TN and AKS performed the qRT-PCR experiments. TN, AV, and SC analysed the data. TN wrote the initial draft. AKS, NPS, AF, DC, AV, and SC revised the manuscript. All authors read and approved the final manuscript.

## Supplementary Information

Below is the link to the electronic supplementary material.

Supplementary file1 (DOCX 446 KB)

## Data Availability

All RNA NGS datasets generated for this study have been deposited in the NCBI Sequence Read Archive (SRA) database (submission ID: PRJNA786755) and can be found here: [https://www.ncbi.nlm.nih.gov/Traces/study/?acc=XXX].

## Abbreviations

VA/VB-S: Tomato leaf curl Gujarat virus*-*infected symptomatic leaves
VA/VB-R: Tomato leaf curl Gujarat virus*-*infected recovered leaves
