# Correction to: Differential expression of genes during recovery of *Nicotiana tabacum* from tomato leaf curl Gujarat virus infection

**DOI:** 10.1007/s00425-023-04206-z

**Published:** 2023-07-25

**Authors:** T. Namgial, A. K. Singh, N. P. Singh, A. Francis, D. Chattopadhyay, A. Voloudakis, S. Chakraborty

**Affiliations:** 1 Laboratory of Plant Breeding and Biometry, Department of Crop Science, Agricultural University of Athens, Athens, 11855 Greece; 2 Molecular Virology Laboratory, School of Life Sciences, Jawaharlal Nehru University, New Delhi, 110067 India; 3 Laboratory of Plant Molecular Biology, National Institute of Plant Genome Research, New Delhi, 110067 India

**Correction to: Planta (2023) 258:37** 10.1007/s00425-023-04182-4

In the original publication, Prof. Supriya Chakraborty is missed to identify as co-corresponding author of an article.

The original article has been corrected.

