# Supplementary material for: Differential expression of genes during recovery of Nicotiana tabacum from tomato leaf curl Gujarat virus infection
Source: Planta. 2023 Jul 5;258(2):37. doi: 10.1007/s00425-023-04182-4 (PMC10322791; doi:10.1007/s00425-023-04182-4)
Supplement: Supplementary file 1 — Supplementary file1 (DOCX 446 KB) [file 425_2023_4182_MOESM1_ESM.docx]

**SUPPLEMENTARY MATERIAL**

**Differential expression of genes during recovery of *Nicotiana tabacum* from tomato leaf curl Gujarat virus infection**

**Namgial T^1,2^, Singh AK^2,#^, Singh NP^3,#^**,**Francis A^3,#^, Chattopadhyay D^3,#^,Voloudakis A^1,*^,Chakraborty S^2,*^**

^1^Laboratory of Plant Breeding and Biometry, Department of Crop Science, Agricultural University of Athens, 11855 Athens, Greece

^2^Molecular Virology laboratory, School of Life Sciences, Jawaharlal Nehru University, 110067 New Delhi, India

^3^Laboratory of Plant Molecular Biology, National Institute of Plant Genome Research, 110067 New Delhi, India

*****Authors for correspondence

**^#^**Authors with equal contribution


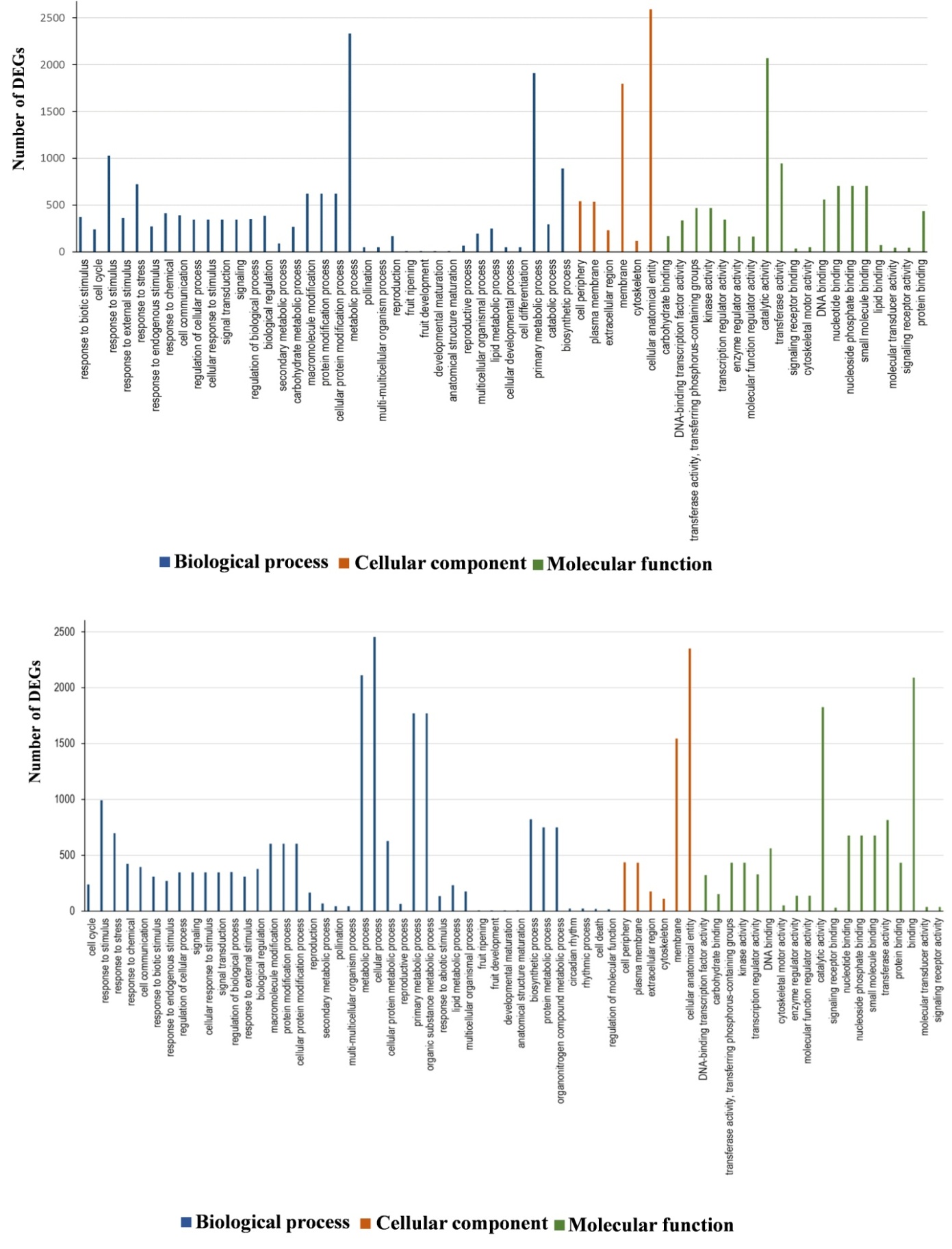


**Fig. S1.** Functional classification of differentially expressed genes (DEGs) using Gene Ontology (GO). **a**) function of DEGs in VA/VB-S (symptomatic leaf) vs. mock-treated plants, **b** VA/VB-R (recovered leaf) plants vs. mock-treated plants.

**Table S1**. List of the forward (F) and reverse (R) primers used for RT-qPCR

| **NAME OF GENE** | **PRIMER NAME** | **PRIMER SEQUENCE**  **(5’ to 3’)** |
| --- | --- | --- |
| *Germin-like protein subfamily T member 2* | NtGLPSTF | CATCAACATCTCTTAACGGCTTTC (Sense) |
|  | NtGLPSTR | CATTGTCTGTGTCTCCCTCTTT (AntiSense) |
| *Cysteine protease inhibitor 1-like* | NtCPILF | CTACTCTCTCTTTGCTTCCCTTT (Sense) |
|  | NtCPILR | ATAGGGAAACCCTTGCCATC (AntiSense) |
| *Thaumatin-like protein* | NtTLPF | CCCATCAGAGCTTCAGGTTAT (Sense) |
|  | NtTLPR | GGGCTCCCATATTTGTTCCT (AntiSense) |
| *Kirola-like* | NtKLF | GTCCCAGATCCACACACTTTA (Sense) |
|  | NtKLR | TGAGACAGCAGCCTAATTCC (AntiSense) |
| *Ethylene-responsive transcription factor ERF109-like* | NtERTFLF | CGAAGAAGAGTTCTGGGATCAA (Sense) |
|  | NtERTFLR | CCTCCGGTAGAATCAGAAGATTT (AntiSense) |
| *Auxin-responsive protein SAUR71-like* | NtARPSLF | GTACGTACCACCTACTTGAACC (Sense) |
|  | NtARPSLR | ATGTCTCGTCGCAAGGAATAG (AntiSense) |
| *Histone H2AX-like* | NtHHLF | TGGCTCTGGTTCACCAATTTA (Sense) |
|  | NtHHLR | GCCTCGGAATTATCCTAGTCTTC (AntiSense) |
| *Uncharacterized* | NtUNCDF | TCAGTTAGAAGTGGAGGGAGAG (Sense) |
|  | NtUNCDR | TTCTCAACAGGGCCAACAC (AntiSense) |
